# Supplementary material for: Sliding induced multiple polarization states in two-dimensional ferroelectrics
Source: Nat Commun. 2022 Dec 13;13:7696. doi: 10.1038/s41467-022-35339-6 (PMC9744910; doi:10.1038/s41467-022-35339-6)
Supplement: Supplementary file 1 — Supplementary Information [file 41467_2022_35339_MOESM1_ESM.pdf]

Supplementary Information

Sliding induced multiple polarization states in two-dimensional ferroelectrics

Peng Meng[1,2,#], Yaze Wu[3,#], Renji Bian[1,#], Er Pan[1,#], Biao Dong[4], Xiaoxu Zhao[5], Jiangang Chen[1], Lishu Wu[6], Yuqi Sun[1], Qundong Fu[6], Qing Liu[1], Dong Shi[1], Qi Zhang[4], Yong-Wei Zhang[3,*], Zheng Liu[6,7,8,*], Fucai Liu[1,2,*]

1 School of Optoelectronic Science and Engineering, University of Electronic Science and Technology of China, Chengdu, China.

2 Yangtze Delta Region Institute (Huzhou), University of Electronic Science and Technology of China, Huzhou, China.

3 Institute of High Performance Computing, Agency for Science, Technology and Research (A*STAR), Singapore, Singapore.

4 School of Physics, Nanjing University, Nanjing, China.

5 School of Materials Science and Engineering, Peking University, Beijing, China.

6 School of Materials Science and Engineering, Nanyang Technological University, Singapore, Singapore.

7 CINTRA CNRS/NTU/THALES, UMI 3288, Research Techno Plaza, Singapore, Singapore.

8 Institute for Functional Intelligent Materials, Nanyang Technological University, Singapore, Singapore.

#These authors contributed equally: Peng Meng, Yaze Wu, Renji Bian, Er Pan.

*E-mail: zhangyw@ihpc.a-star.edu.sg, z.liu@ntu.edu.sg, fucailiu@uestc.edu.cn

Supplementary Figures and Tables

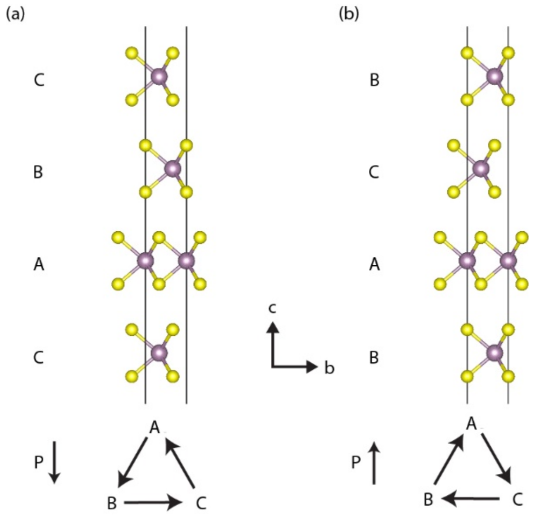

Fig. S1 Notation for the relative positions of each layer with respect to other layers. The series begins from the bottom layer to the top. Polarization is switched when the layer stacking order is switched from cyclic (a) to anticyclic (b).

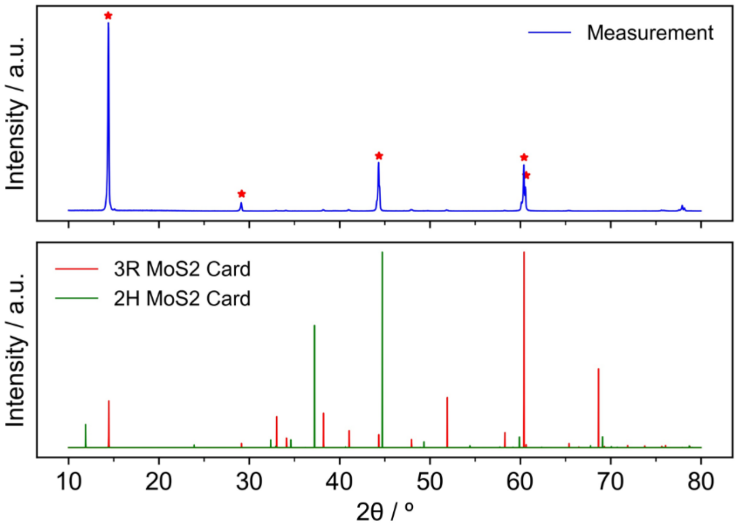

Fig. S2. Powder XRD patterns of 3R MoS_2 (upper panel) and the ICDD card for 3R and 2H MoS_2 (lower panel). The crystal is ground to powder before XRD measurement.

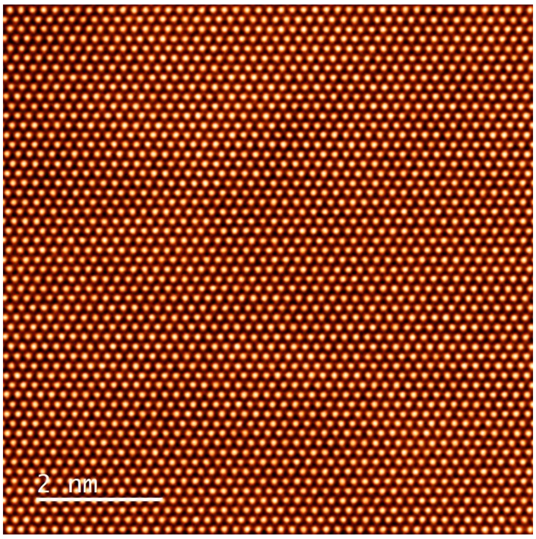

Fig. S3. Atomic-resolution ADF-STEM images of 3R MoS_2. See Supplementary Note 1 for detailed discussions.

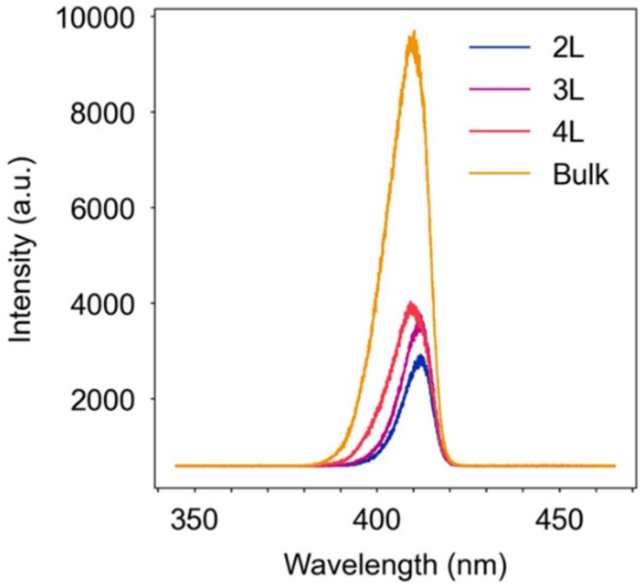

Fig. S4 SHG spectra obtained from 3R MoS_2 of different layer numbers, measured at room temperature.

Table S1. Parameters of 3R MoS_2 devices.

Device	Top BN (nm)	Bottom BN (nm)	A
3R-Bilayer (FET)	13.5	13.8	-0.925
3R-Trilayer (FET)	12.9	12.9	-0.945
3R-Tetralayer (FET)	12.2	14.7	-1.065
2H-Bilayer (FET)	12.8	12.6	-1
3R-Tetralayer (FTJ)	28.1	29.1	-1

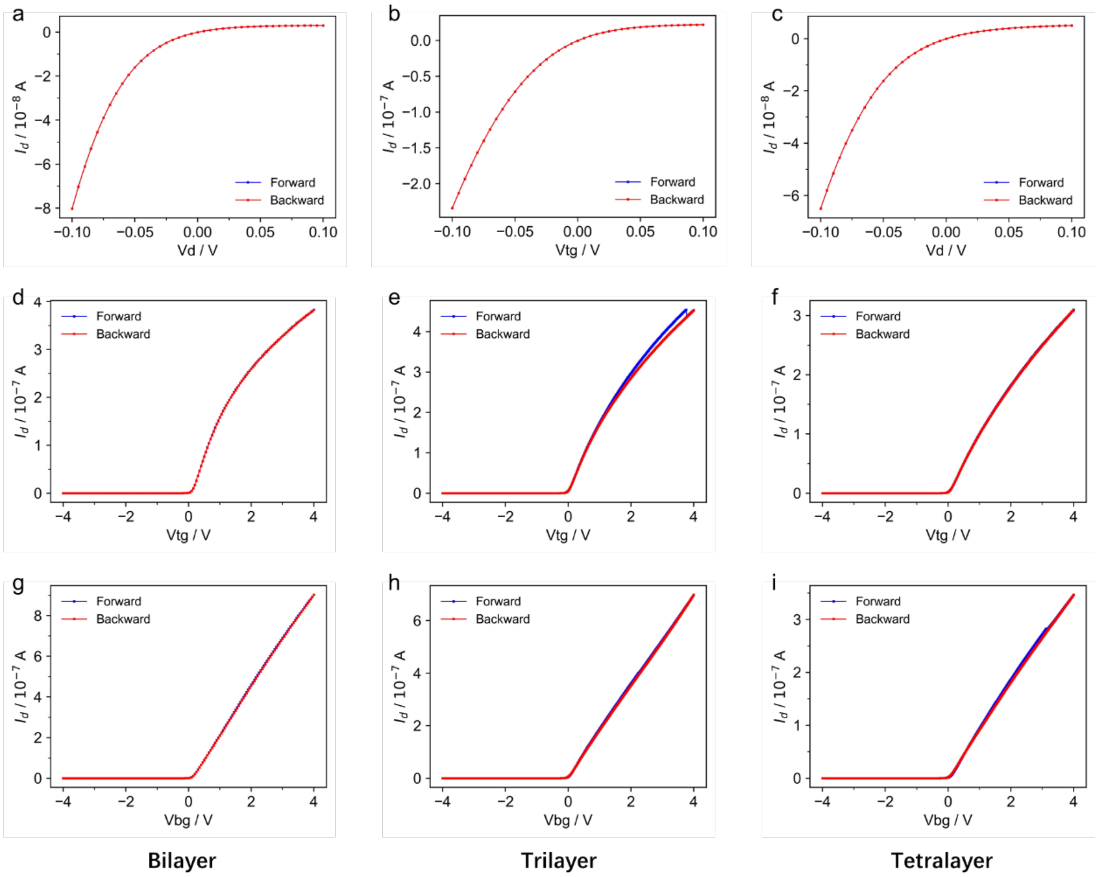

Fig. S5. The I_d-V_d, I_d-V_{tg} and I_d-V_{bg} plots of **(a)(d)(g)** bilayer, **(b)(e)(h)** trilayer and **(c)(f)(i)** tetralayer devices.

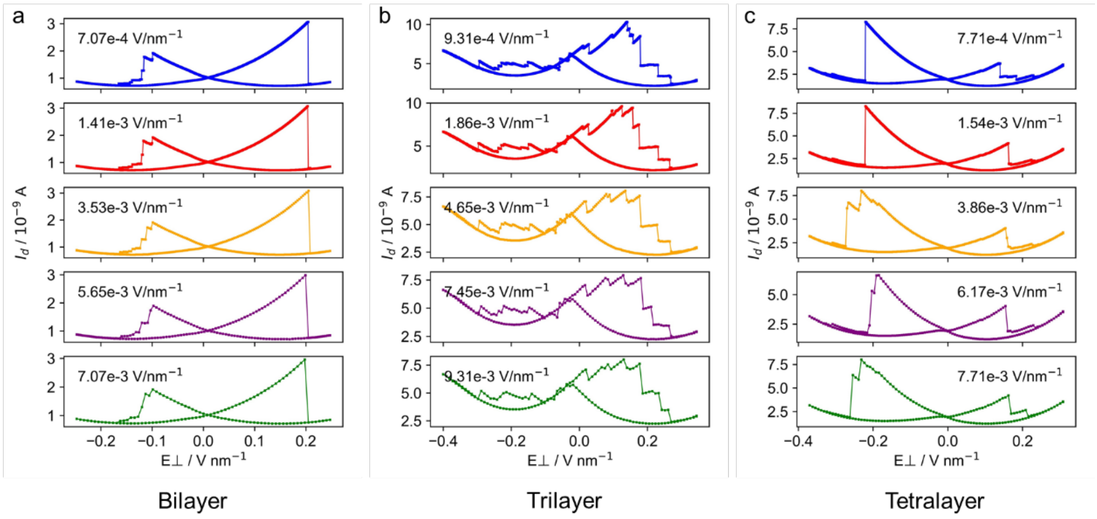

Fig. S6. I_d as a function of $E_\perp$ at different sweeping rates in **(a)** bilayer, **(b)** trilayer and **(c)** tetralayer devices.

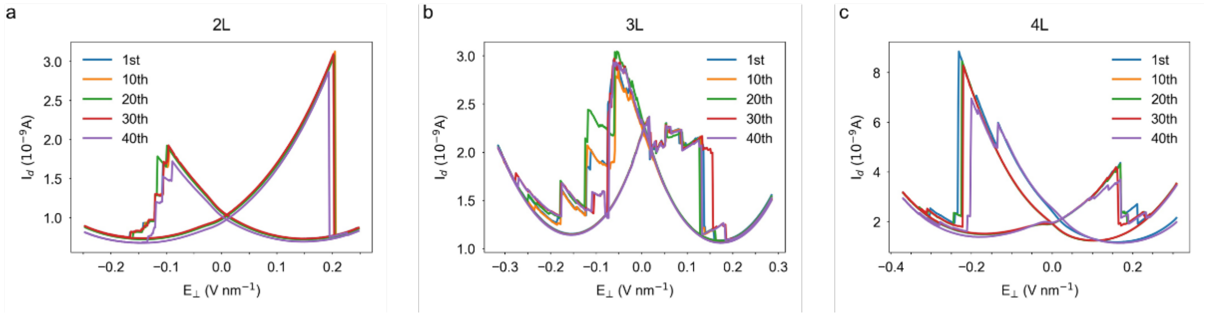

Fig. S7 The cycling performance for dual-gate 3R MoS_2 FET devices with different thickness in 40 cycles. (a) Bilayer, (b) trilayer, (c) tetralayer.

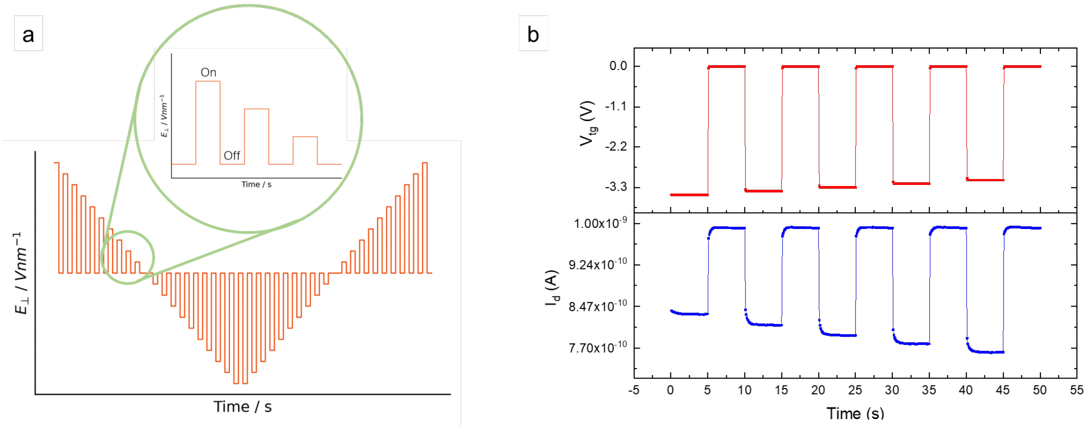

Fig. S8. (a) The overall plot of triangular electric field waveform. Voltage amplitude sweeping, through the voltage train with a duration of 5s (on-field) and time interval of 5s (off-field), is applied to the device. (b) The data of V_{tg} and I_d in the static transport measurement for a 50s time period. Both the "on-field" and "off-field" segments are 5s. The upper panel shows the voltage on top gate (V_{tg}), and the bottom gate voltage (V_{bg}) is defined by $V_{bg} = AV_{tg} + B$ as discussed in the manuscript. The lower panel shows the I_d under monitoring.

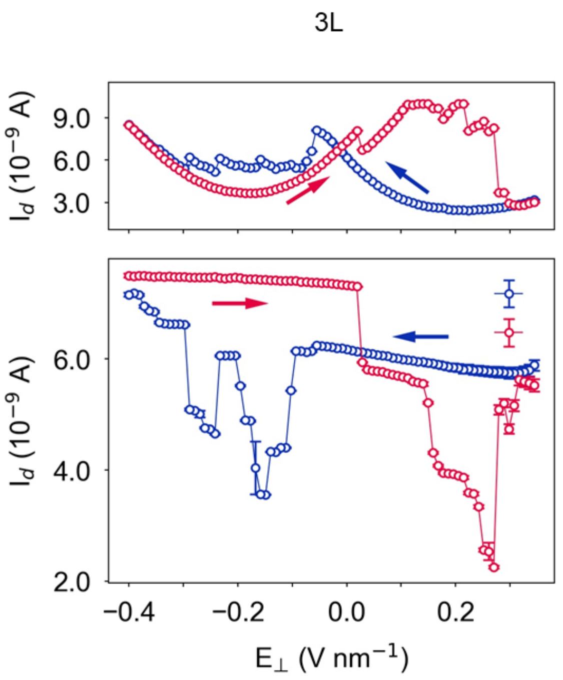

Fig. S9 The averaged I_d as a function of $E_\perp$ in on-field (upper panel) measurements and off-field (lower panel) measurements in dual-gated FET device of an additional trilayer 3R MoS_2 device. The result is similar to that shown in Fig. 3b, even though slight variation exists. These variations maybe caused by stress introduced during fabrication.

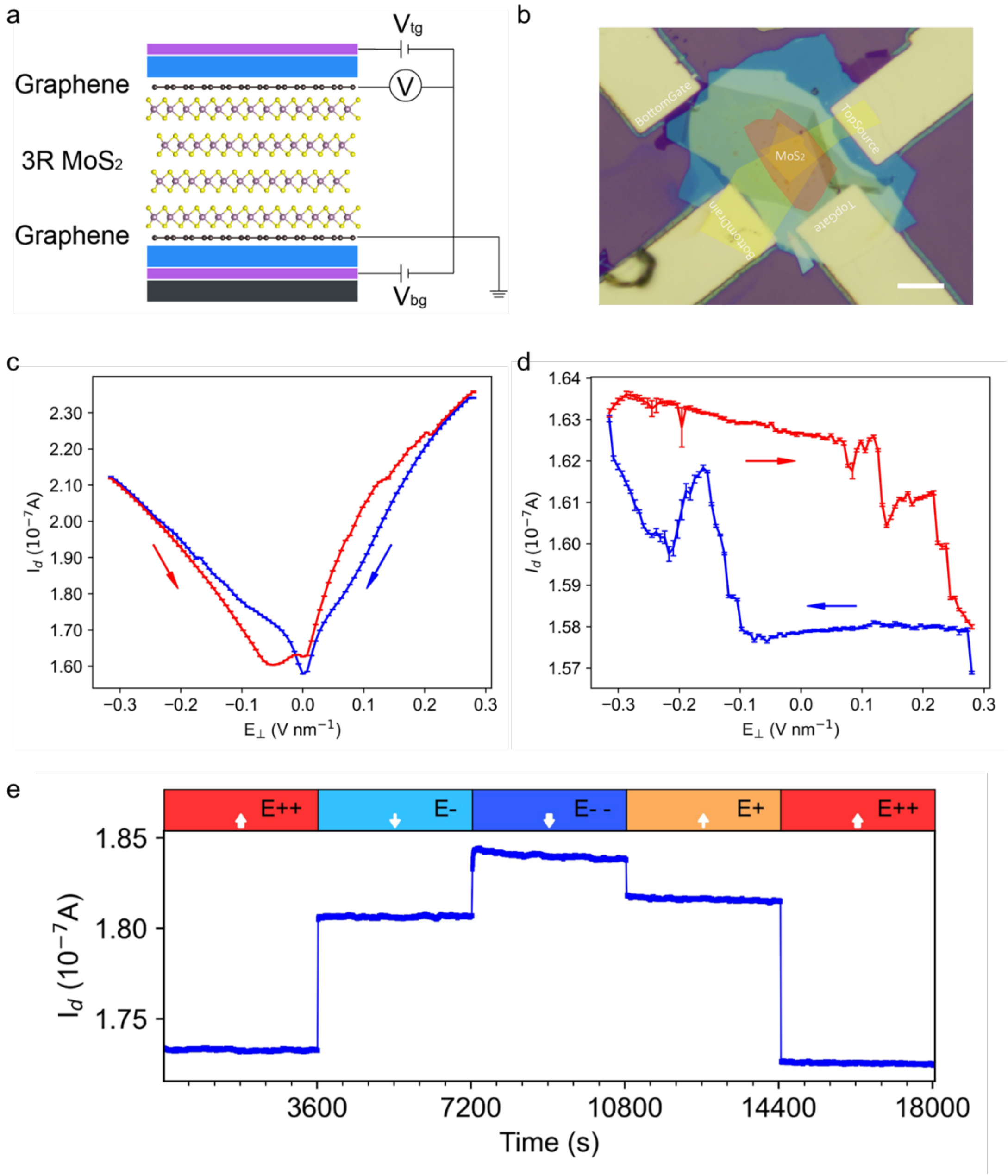

Fig. S10. Dual-gate ferroelectric tunnelling junction (FTJ) based on tetralayer 3R MoS_2. **(a)** Schematic of the FTJ device structure. The blue blocks represent h-BN, the purple blocks represent the graphite gates, and the grey block represents the SiO_2(285 nm)/Si substate. **(b)** Micrograph image of the FTJ device. The scale bar is 10 µm. Averaged I_d as a function of the triangular electric field waveform ($E_\perp$) in **(c)** on-field sweep and **(d)** off-field sweep. **(e)** Retention of different states for 1h in FTJ.

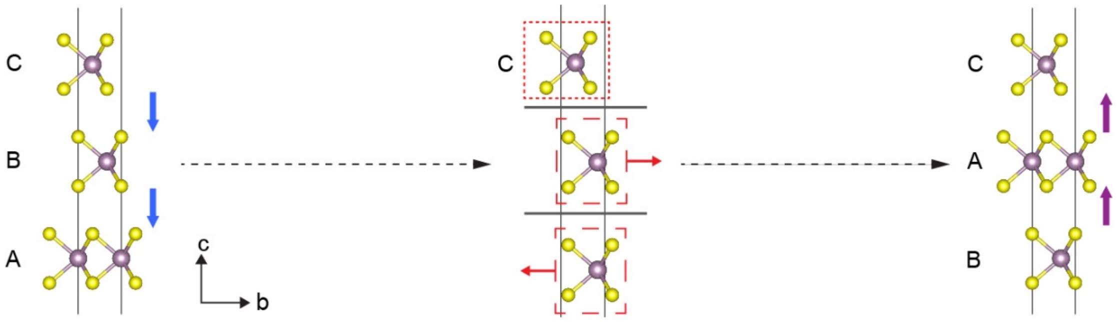

Fig. S11 Variation to ferroelectric switching Path 3 for trilayer 3R MoS_2.

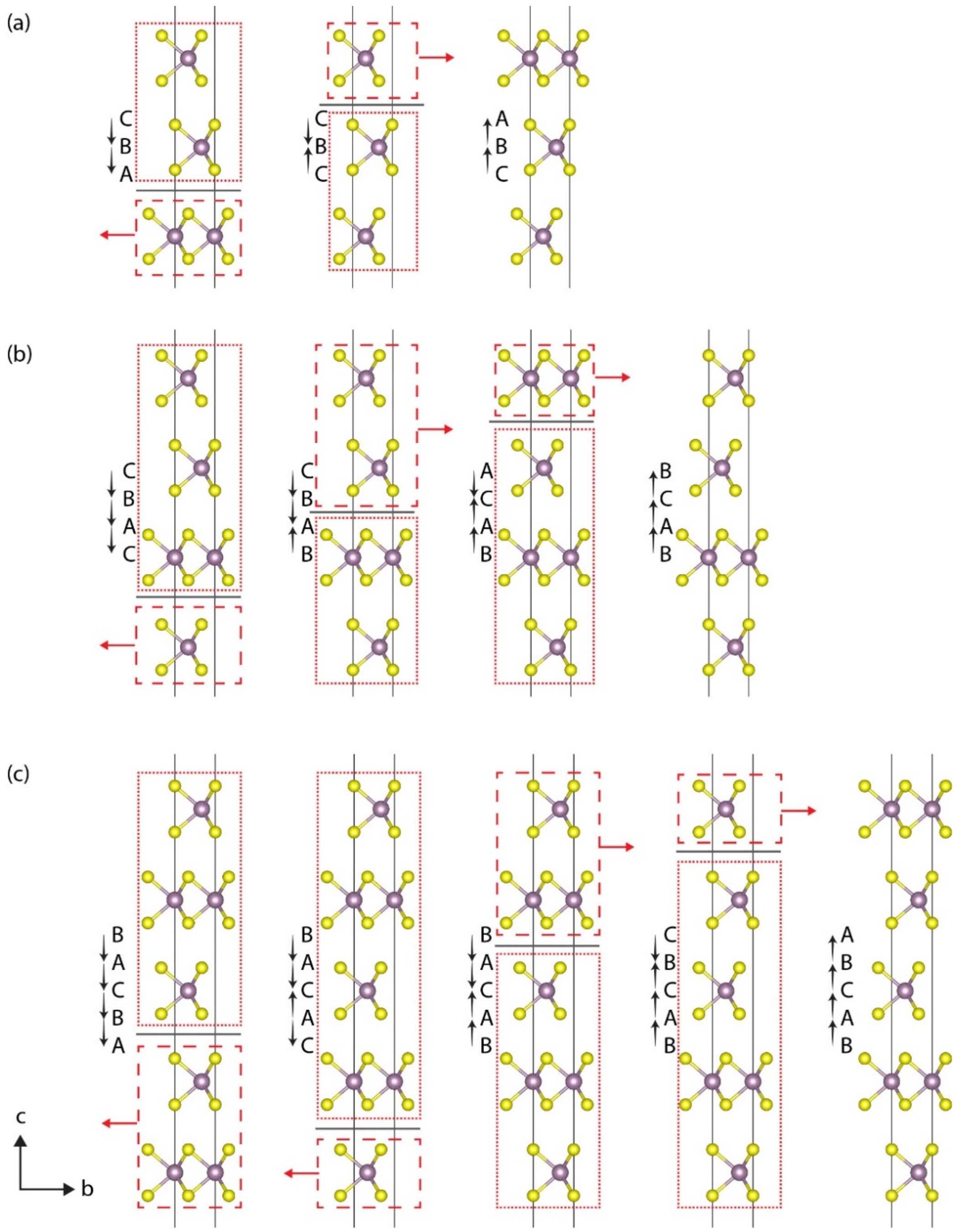

Fig. S12. Atomic structures of stable intermediate polarization states produced by the generalized model for **(a)** trilayer, **(b)** tetralayer and **(c)** pentalayer 3R MoS_2. Red dashed boxes mark out the moving blocks. Red horizontal arrows indicate the blocks' directions of translation immediately after the state shown. Red dotted boxes mark out the stationary blocks. Each black vertical arrow indicates the direction of interface dipole between the layers sandwiching the arrow. Each set of black vertical arrows also illustrates the different polarization configurations in each state.

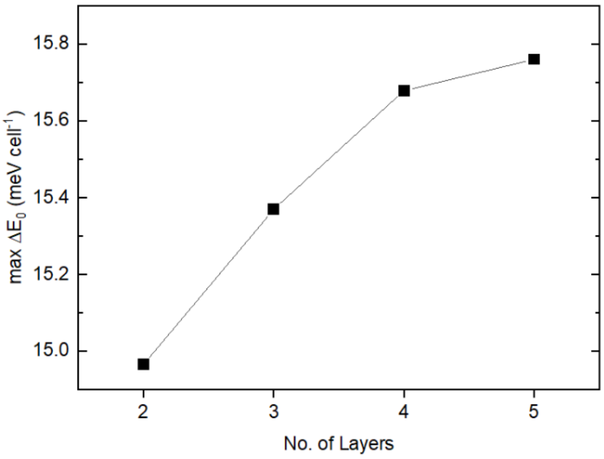

Fig. S13. Relationship between maximum energy barriers of the generalized model in n-layer 3R MoS_2 and number of layers (n).

Supplementary Note 1

TEM Results: The atomic resolution STEM-ADF image shows a hexagonal lattice as marked by the white diamond box (Fig. 1b), which is consistent with the schematic in Fig. 1a. The d-spacing of (110) plane is measured to be 1.5 Å. Since each layer in 3R phase is relatively shifted by a step of 1/3 unit cell in the direction of $(\mathbf{b}-\mathbf{a})$, each Mo atom is always aligned with two S atoms in $\boldsymbol{c}$-axis direction (Fig. 1a). This atomic eclipse makes it impossible to differentiate the type of atoms using the STEM image contrast variations. The reverse is true in 2H MoS_2. The FFT patterns are shown in the inset of Fig. 1b, where a six-fold symmetry is clearly observed.

Supplementary Note 2

Carrier Density Estimation: By fitting the linear slope of I_d-V_{bg} plot in Fig. S5g, the threshold voltage (V_{th}) of the bottom gate in bilayer 3R MoS_2 dual-gate FET is extrapolated to be 0.136 V. $V_{bg}=0.6\ V$ is substituted to the formula: $n=\frac{\varepsilon_r\varepsilon_0 S(V_{bg}-V_{th})}{dq}$, where ε_r is the relative permittivity of h-BN (~4.0), ε_0 is the absolute permittivity of vacuum (8.85×10^{-12} F m^{-1}), S is the area of the channel (37.58 nm^2), d is the thickness of the channel (13.78 nm) and q is the value of elementary charge (1.6×10^{-19} C). The carrier density at V_{bg} of 0.6 V is estimated to be 3.6×10^{14} m^{-2}.

Supplementary Note 3

Dual-gate FTJ results: The tetralayer 3R MoS_2 is also implemented in a ferroelectric tunnelling junction (FTJ). As shown in Fig. S10a, the FTJ is fabricated in a dual-gate setup with monolayer graphene as the top and bottom electrodes. Due to the limited density of states (DOS) in monolayer graphene, the electric field can penetrate the entire junction. The optical image of the device is shown in Fig. S10b. To investigate the switching process in the FTJ, the triangular waveform electric field illustrated in Fig. 3a is applied. In the on-field measurement, due to

the minimization of the carrier density variation in 3R MoS_2 by dual-gate regulation, the transport property of the FTJ is determined predominantly by the graphene layer and the 3R MoS_2 acts as an embedded dielectric layer. As shown in Fig. S10c, a clear hysteresis caused by the ferroelectric switching of 3R MoS_2 can be observed. In each sweep direction, the curve is the combination of transport curves from the two graphene layers under the sweeping gate voltages. In the off-field measurement, the curve reflects the intrinsic property of the junction. Because of the small reading voltage (1 mV) and the semimetal nature of graphene, the resistance of the junction mainly originates from the 3R MoS_2. In Fig. S10d, a hysteresis window is observed, consistent with results from other FTJ reports[1, 2]. This again confirms the ferroelectricity in 3R MoS_2. In conventional FTJ, during the ferroelectric switching, the flipping of dipoles and the evolution of domain boundaries exhibit monotonous change of tunnelling current[3]. However, in tetralayer 3R MoS_2, we observe the formation of intermediate states as shown in Fig. S10d. Combined with observations made in Fig. 3, we see that occurrence of the intermediate states is independent of the current flow direction in 3R MoS_2. It is hence appropriate to conclude that there is a new dimension, i.e. the interlayer dipole coupling, to controlling the sliding ferroelectricity in multilayer (more than bilayer) 3R MoS_2. Finally, the retention of the FTJ in different states is also studied. As shown in Fig. S10e, all the states are very stable in 1 h, which confirms again that the intermediate states are energetically stable. The stability of the multiple polarization states will benefit the practical applications in the future.

Supplementary Note 4

Polarization Reversal in 3R MoS_2: The source of spontaneous polarization in 3R MoS_2 can be traced to the asymmetric accumulation of electronic charge in the out-of-plane direction. This is due to the charge transfer between sulphur atoms of adjacent atomic layers across the vdW gap. The direction of charge transfer follows the cyclic stacking order of the atomic layers such that electrons are transferred from the layer earlier in the cyclic

order to the layer later in the order. We illustrate this using the bilayer 3R MoS_2. In the initial state of the ferroelectric switching process (see Fig. S14, 0%), the bottom and top atomic layers have stacking positions B and C, respectively, following the cyclic order of …A<u>BC</u>AB… The electrons are transferred from the B layer to the C layer, following the cyclic order. The accumulation of electrons in the C layer in turn gives rise to the downward spontaneous polarization in the initial state. As the top atomic layer translates from C to A to reach the final state (Fig. S14, 100%), electrons are progressively transferred from the top (A) layer to the bottom (B) layer (see Fig. S15a), resulting in a reversed spontaneous polarization compared to the initial state and hence demonstrating out-of-plane ferroelectricity switching.

The cyclic order-dependent charge transfer between the sulphur atoms of adjacent atomic layers can be attributed to the interactions between their p_z orbitals. In Fig. S14, wavefunctions taken at the electronic state with large sulphur p_z orbital projection (Γ point of the highest valence band, see Fig. S16 for details) show different sizes of wavefunction isosurfaces on the sulphur atoms in the initial and final states. This agrees with the charge transfer observed in Fig. S15a. Moreover, the position of electron accumulation corresponds well with the direction of spontaneous polarization. This cyclic order-dependent charge transfer can be generalized to describe the polarization reversal in 3R MoS_2 of any number of layers (See Fig. S15b-d).

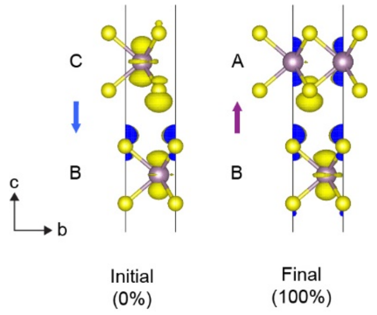

Fig. S14 Real space distribution of electronic wavefunctions at the Γ point of the highest valence band in the initial and final ferroelectric states of bilayer 3R MoS_2. Atomic structures of the two states are also embedded. The isosurfaces are extracted at $4 \times 10^{-8}\ e\ Bohr^{-3}$. Cross-sections of the wavefunctions are coloured in blue. The directions of spontaneous polarizations are marked by the blue (downward polarization) and purple (upward polarization) arrows.

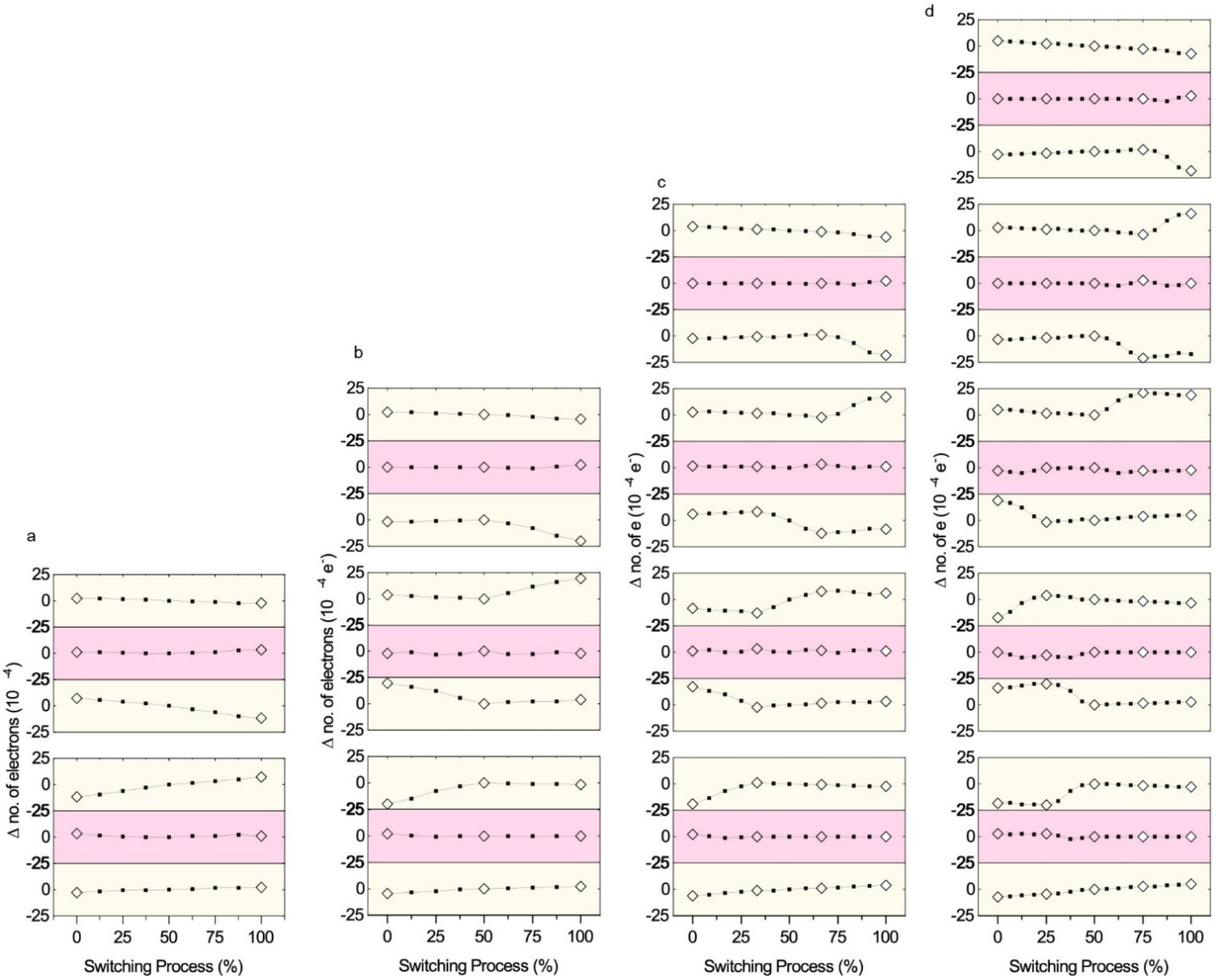

Fig. S15 Evolution of Bader charges in n-layer 3R MoS_2 during the ferroelectric switching process from cyclic stacking (i.e. AB, ABC, CABC, ABCAB) to anticyclic stacking (i.e. BA, CBA, BACB, BACBA). Presented here are results for **(a)** bilayer, **(b)** trilayer, **(c)** tetralayer and **(d)** pentalayer. The number of electrons at the midpoint (50%) of the switching process is taken as zero reference. The beige and magenta rows denote the sulphur and the molybdenum atoms, respectively. The pathways corresponding to b, c and d can be found in Fig. S12.

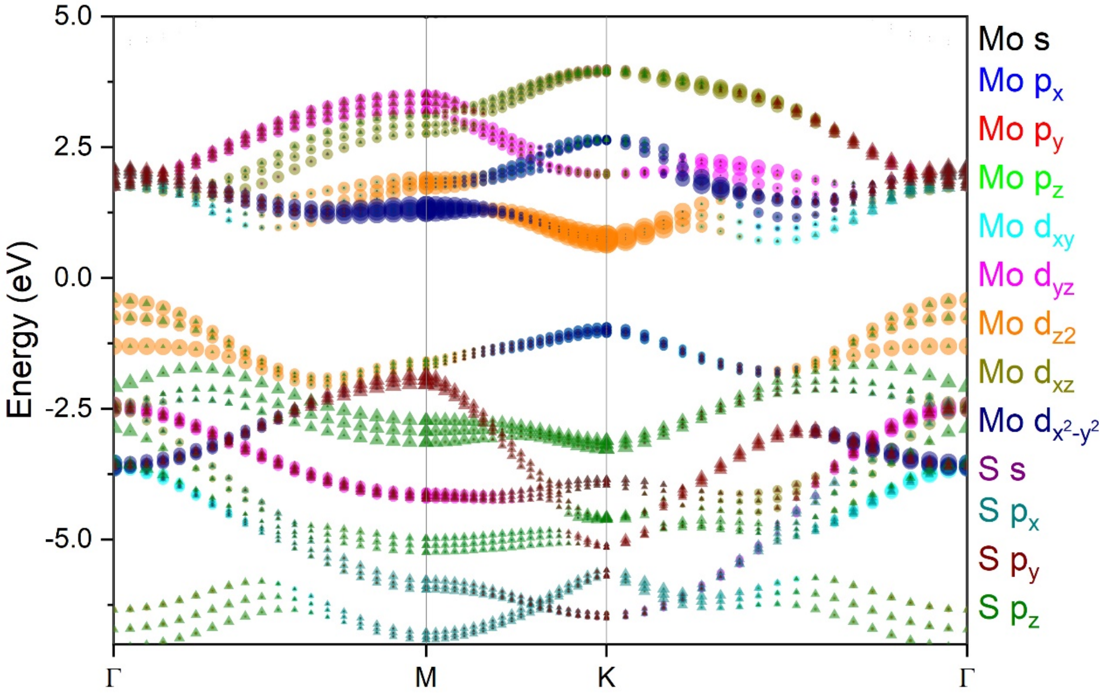

Fig. S16. Orbital projected DFT band structure. Circles denote Mo orbitals and triangles denote S orbitals. The size of the symbols denotes the size of projection.

Supplementary Note 5

Potential Energy Surface Calculation Results: To identify the minimum energy path (MEP) among the various pathways proposed for a trilayer 3R MoS_2, the potential energy surface (PES) experienced by the translating atomic layers in all possible switching pathways are exhaustively mapped. Here, we define the interaction energy among the atomic layers in any arbitrary stacking order as $E_i = E_{123} - E_{1+2+3}$, where E_{123} is the total energy of the trilayer while E_{1+2+3} is the sum of the total energies from each of the three atomic layers (1, 2 and 3) isolated from one another. The PES experienced by the translating atomic layer(s) is obtained by taking 9 evenly spaced samples of E_i from the initial position of its(their) Mo atom(s), along the direction of its(their) translation $\pm(\mathbf{b} - \mathbf{a})$, until the nearest periodic image of the initial atomic structure is encountered. Because of symmetry in the 3R MoS_2 hexagonal unit cell, sampling along this route is equivalent to sampling the edges of the lattice irreducible zone[4].

Supplementary Note 6

Origin of Energy Barriers in 3R MoS_2: To understand the superior thermodynamic performance of Path 1 compared to other paths, we investigate the origin of the energy barriers in all the paths. As shown in Fig. S17a and b, the energy profiles of the paths show close resemblance to their respective thickness profiles. It is noted that there is negligible change in the thickness of each S-Mo-S atomic layer during the ferroelectric switching process, hence all changes in thickness of the trilayer 3R MoS_2 arise from changes in the vdW gap. This suggests that the energy barriers originate from electronic repulsion between the atomic layers. Furthermore, the size of vdW gap between two adjacent layers is independent from the movements or stacking orders of other layers, as shown in Fig. S17c. This suggests that the interlayer repulsion is short ranged and hence limited to the pair of layers that experiences the translation relative to each other.

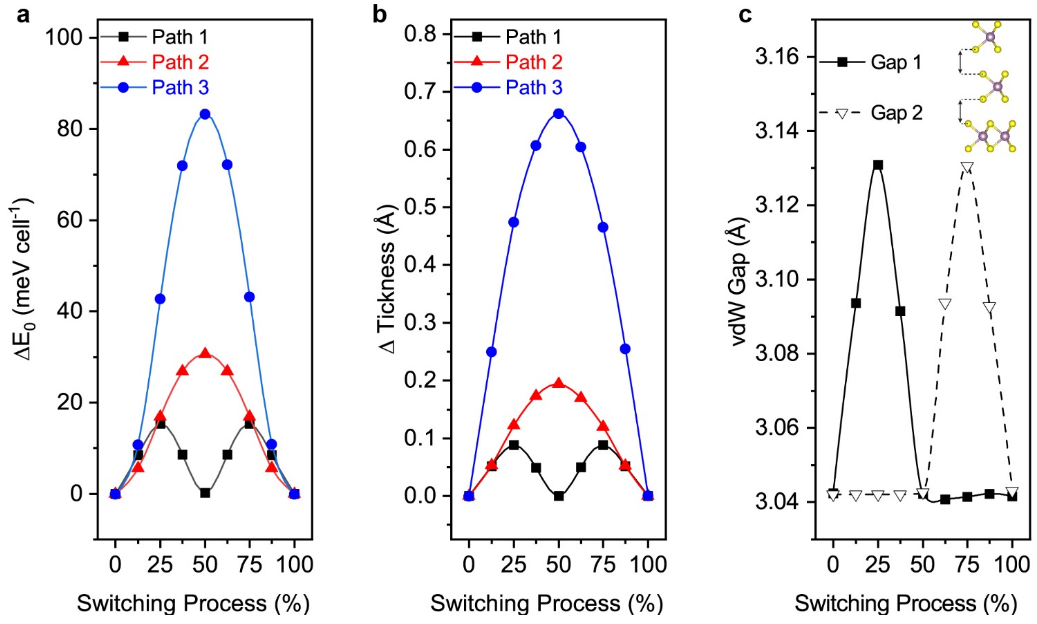

Fig. S17 Changes during the ferroelectric switching process. **(a)** Change in total energy of trilayer 3R MoS_2 in Path 1-3. **(b)** Change in thickness of trilayer 3R MoS_2 in Paths 1-3. Thickness here is defined as the distance in the z-direction between the topmost and bottommost atoms in the trilayer 3R MoS_2. **(c)** Height of the vdW gaps of trilayer 3R MoS_2 in Path 1. Inset: illustration of the gaps.

Supplementary Note 7

Ferroelectric switching in a defect system: To evaluate the effect of defects on the ferroelectric switching process in 3R MoS_2, a $6 \times 6 \times 1$ unit cell trilayer 3R MoS_2 with a S vacancy on the topmost sulphur layer is used to replicate the most likely defect occurring in MoS_2 systems. This replicates a defect concentration of 0.46%.

The S vacancy is observed to lift the energy degeneracy of barriers from the ABC-CBC and ACB-ABA processes of Path 1. The barrier of the ABC-CBC transition shows a higher energy barrier than the ABC-ABA process, which involves the translation of the atomic layer with the S vacancy. Subsequently, the CBC-CBA process, which involves the translation of the atomic layer with the S vacancy, shows a smaller energy barrier than the ABA-CBA process (see. Fig. S18) Hence the ABC-ABA process in this case is always energetically favourable compared to the ABC-CBC process. Similarly, the CBC-CBA process is always energetically favourable compared to the ABA-CBA process. Consequently, the ABC-ABA-CBA process is always preferred in the ramp-up process of this defect system, and the CBA-CBC-ABC process is always preferred in the ramp-down process. In the actual experiment, where defects are inevitable, the switching process with the lower initial energy barrier is always preferred. This agrees well with our experimental observations where identical $I_d - E_\perp$ relationship is observed even after 40 cycles.

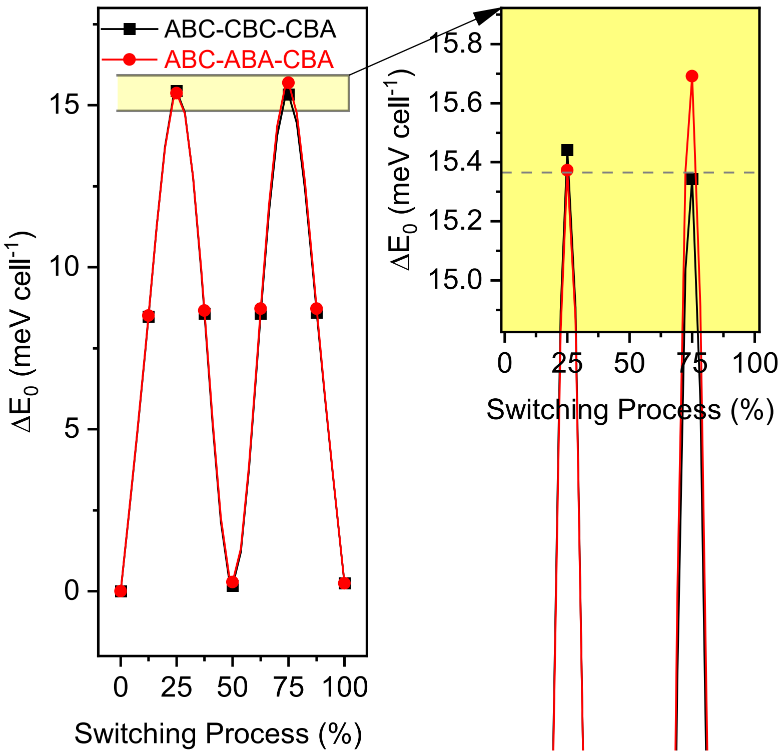

Fig. S18 Energy barriers of Path 1 when a S vacancy is present in the top atomic layer. The total defect concentration of the system is 0.46%. The inset zooms in on the contents of the yellow box in the main figure. The dashed horizontal line marks the height of energy barriers in the pristine case.

Despite the changes in the energy barriers, the difference in out-of-plane spontaneous polarizations between each corresponding polarization states of the two defect processes is negligible (see Fig. S19). This difference between the defective system and pristine system is also very small (see Fig. S19). As the 0.46% defect concentration is larger than what is usually present in experiment, the real-life change in spontaneous polarization in 3R MoS_2 should not be significantly affected by S vacancy.

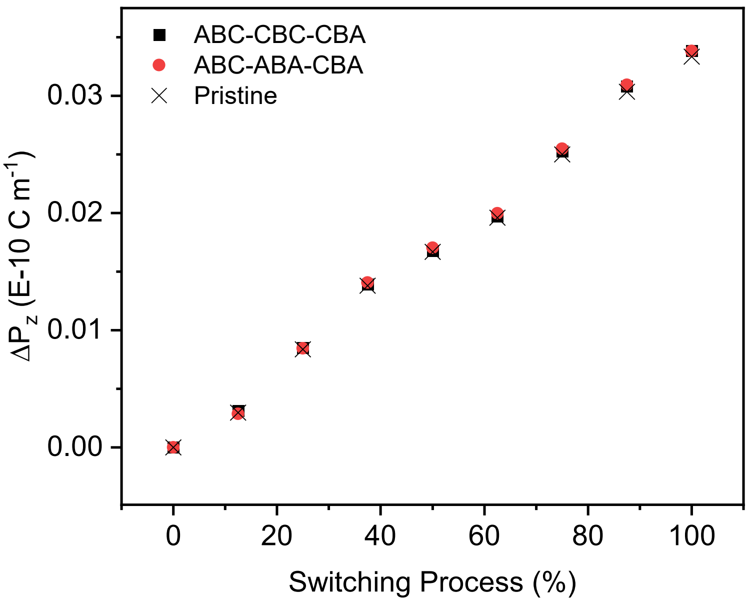

Fig. S19 Change in out-of-plane spontaneous polarizations of various states in Path 1 when a S vacancy concentration of 0.46% is considered. The spontaneous polarization of the initial state is taken as zero reference.

Supplementary References

1. Garcia V, Bibes M. Ferroelectric tunnel junctions for information storage and processing. *Nature Communications* **5**, 4289 (2014).
2. Wen Z, Wu D. Ferroelectric Tunnel Junctions: Modulations on the Potential Barrier. *Adv Mater* **32**, 1904123 (2020).
3. Chanthbouala A*, et al.* Solid-state memories based on ferroelectric tunnel junctions. *Nature nanotechnology* **7**, 101-104 (2011).
4. Levita G, Cavaleiro A, Molinari E, Polcar T, Righi MC. Sliding Properties of MoS2 Layers: Load and Interlayer Orientation Effects. *The Journal of Physical Chemistry C* **118**, 13809-13816 (2014).
